# Left Ventricular Diastolic Function in Hypertension: Methodological Considerations and Clinical Implications

**DOI:** 10.14740/jocmr2050w

**Published:** 2014-12-29

**Authors:** Pasquale Palmiero, Annapaola Zito, Maria Maiello, Matteo Cameli, Pietro Amedeo Modesti, Maria Lorenza Muiesan, Salvatore Novo, Pier Sergio Saba, Pietro Scicchitano, Roberto Pedrinelli, Marco Matteo Ciccone

**Affiliations:** a ASL Department of Cardiology, Brindisi District, Italy; b Cardiovascular Disease Section, Department of Emergency and Organ Transplantation, University of Bari, Italy; c Department of Cardiovascular Diseases, University of Siena, Italy; d Department of Clinical and Experimental Medicine, University of Florence, Florence, Italy; e Clinica Medica, Department of Clinical and Experimental Sciences, University of Brescia, Brescia, Italy; f Department of Internal Medicine and Cardiovascular Diseases, Palermo, Italy; g Cardiologia, Azienda Ospedaliero-Universitaria di Sassari, Italy; h Dipartimento di Patologia Chirurgica, Medica, Molecolare e dell’Area Critica, Universita di Pisa, Pisa, Italy

**Keywords:** Left ventricular, Diastolic function, Diastolic dysfunction, Hypertension

## Abstract

The assessment of left ventricular (LV) diastolic function should be an integral part of a routine examination of hypertensive patient; indeed when LV diastolic function is impaired, it is possible to have heart failure even with preserved LV ejection fraction. Left ventricular diastolic dysfunction (LVDD) occurs frequently and is associated to heart disease. Doppler echocardiography is the best tool for early LVDD diagnosis. Hypertension affects LV relaxation and when left ventricular hypertrophy (LVH) occurs, it decreases compliance too, so it is important to calculate Doppler echocardiography parameters, for diastolic function evaluation, in all hypertensive patients. The purpose of our review was to discuss about the strong relationship between LVDD and hypertension, and their relationship with LV systolic function. Furthermore, we aimed to assess the relationship between the arterial stiffness and LV structure and function in hypertensive patients.

## Introduction

**Figure 1:**
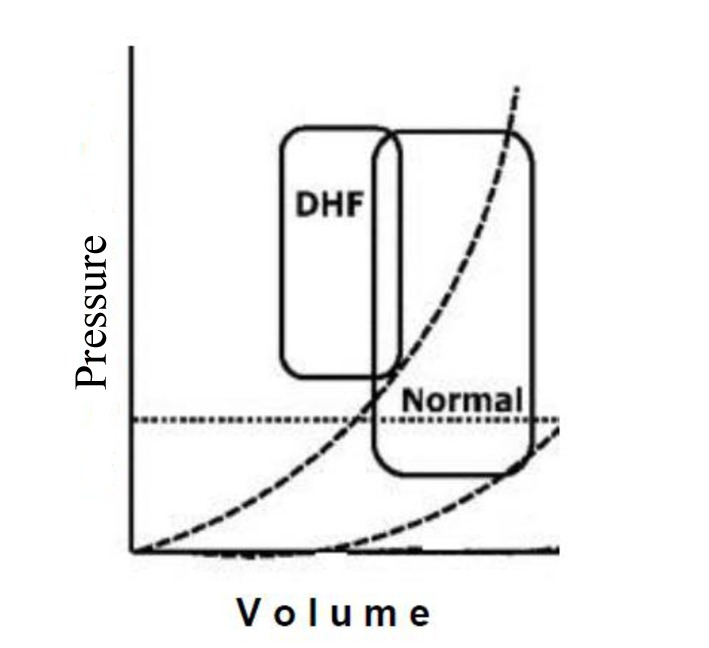
Pressure/volume loop in LVDD.

Left ventricular diastolic dysfunction (LVDD) is an earlier alteration common to many cardiovascular diseases [1, 2]. Any kind of heart disease that leads to myocardial structural alteration and/or pericardial effusion may cause LVDD [1-3]. Sometimes this structural abnormality is evident macroscopically (i.e. hypertrophy, fibrosis, infiltrative diseases, dilated cardiomyopathy, myocardial infarction, constrictive pericarditis, etc.). Other times, diastolic dysfunction is linked to abnormalities of the cellular mechanisms of myocyte relaxation caused by hypoxia and/or ischemia [3]. A lot of patients with congestive heart failure, symptomatic, have a normal left ventricular ejection fraction and signs/symptoms mainly due to diastolic dysfunction [4, 5]. For these reasons, the assessment of LV diastolic function should be always performed during a routine echocardiographic examination, since when LV diastolic function is impaired, it is possible to have heart failure even with preserved LV ejection fraction (HFpEF). Diastolic dysfunction can display a wide spectrum of different patterns, ranging from a simple slowing of ventricular relaxation, without significant hemodynamic changes, to the development of pulmonary venous congestion, due to elevation of ventricular diastolic pressures with displacement of the pressure-volume loop in the upper and to the left [2, 3] (Fig. 1). However, patients affected by heart failure often presents both systolic and diastolic ventricular dysfunction, since the systole and diastole are related one to the other in a complex way. Often changes in diastolic function do not affect only the filling of the ventricles, but also ventricular systolic function [6]. Many factors can influence the ventricular diastolic filling and then the diastolic pressure-volume relationship: coronary insufficiency [7], vasodilators and vasoconstrictors [8] (i.e. changes in preload and after load), geometric-mechanical interactions between the ventricles (e.g. reverse Bernheim effect in pulmonary embolism) [9], pericardial anatomy [10] (e.g. constrictive pericarditis, pericardial effusion), metabolic alterations (acidosis, alkalosis) [11], cardiovascular drugs [12] (e.g. digital, beta-blockers, calcium channel blockers), and hypoxia [13].

**Figure 2:**
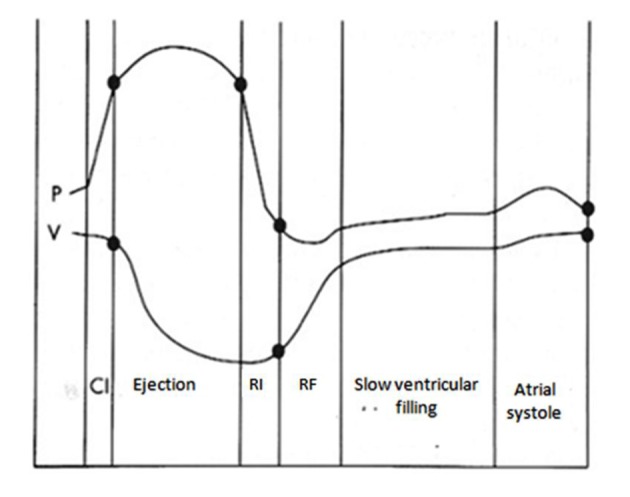
Diastolic phases related with changes in pressure (P) and volume (V) during a cardiac cycle. CI: isovolumic contraction; RI: isovolumic relaxation; RF: rapid filling.

To better understand how LVDD occurs, we have to start from physiopathology of LV diastole. It can be divided into four phases, starting from aortic valve closure. The first phase of diastole is the isovolumic, which does not contribute to ventricular filling. The second of early and rapid filling provides most of ventricular filling, about 60-90%. The third of slow filling, mentioned as diastasis, contributes to only 5% of the total filling. The final atrial booster phase normally accounts for the remaining 5-35%, according to the age, with an increasing contribution in elderly [14] (Fig. 2).

“Isovolumic relaxation time” starts with the closure of the semilunar valves and ends with the opening of the atrioventricular ones. During this phase, there is no change in intraventricular volume (because the valves are closed), but the geometric configuration of the ventricular cavity changes, followed by a fall in ventricular pressure that becomes lower than atrial pressure. Then atrioventricular valve opens and it begins the second phase of diastole: fast ventricular filling [15]. The isovolumic relaxation is an energy-dependent process, where the calcium ions are removed from the cytoplasm against a concentration gradient, allowing the dissociation of the contractile complex actin-myosin (active relaxation) [16]. It can be influenced, not only by the anatomical and functional heart conditions, but even from pre-load and post-load. The speed of relaxation also influences the speed of fall of left ventricular pressure. An impaired relaxation causes an increase in filling pressure to maintain an adequate diastolic volume of the ventricle. Even the “elastic recovery” (the release of elastic energy “compressed” in the myocardium during the previous systole and then released as soon as the relaxation begins) acts on the isovolumic relaxation. Also this release contributes to the fall of left ventricular pressure at the beginning of diastole. A reduction in systolic function, resulting in an increase in left ventricular end-systolic volume, causes a reduction of the “elastic return” in the subsequent early diastole. The gold standard measurement of relaxation rate is dP/dtmax, but it requires invasive catheterization [17]. Similarly, tau, the time constant of relaxation, describes the rate of LV pressure decrease during isovolumic relaxation but it requires invasive techniques [18-20]. On clinical practice, tau is assessed by echocardiography. Tau is increased as the systolic LV pressure increases. The isovolumic relaxation time can be measured by Doppler echocardiography: it lies between aortic valve closure and mitral valve opening, but it suffers of an high intra- and inter-operator variability, so the range of normality is wide [20].

“Ventricular fast filling time” starts with the opening of the atrioventricular valve, proceeds with increasing speed up to a maximum peak, subsequently decelerates, and ends with the beginning of the third phase. During this phase, the blood which had accumulated in the atrium while the valve was closed is poured rapidly into the ventricle, consequently the atrial pressure falls while the ventricular pressure increases. The contribution of rapid ventricular filling to total diastolic one, in healthy adults, is about 65-80% [21]. “Fast ventricular filling time” is a mainly passive process, due to the atrioventricular gradient, that is greatly influenced by ventricular compliance. This phase of diastole is also influenced by the “elastic return” of the ventricle. In the “slow ventricular filling”, the flow of blood from the atrium to the ventricle is very slow, because the pressure gradient atrioventricular is virtually absent. The duration of this phase is greatly dependent on the heart rate [18-21]. “Atrial systole” is the final phase of diastole, present only on sinus rhythm. Atrial active contraction causes an increase of blood flow from the atrium to the ventricle, consequently an increase in pressure and volume occurs and the end-diastolic ventricular activation of Starling’s effect happens [4]. In normal subject, the contribution of atrial systole to the total LV filling is about 20%. An increase in heart rate reduces the contribution of atrial systole. In arterial hypertension, LV filling abnormalities can be detected early and also, often, they precede the impairment of systolic function of the left ventricle. In all phases of diastole, hypertension affects relaxation and when left ventricular hypertrophy (LVH) occurs, it reduces compliance too, so it is important to calculate Doppler echocardiography parameters, for diastolic function evaluation, in all hypertensive patients. The goal of our paper is to assess the strong relationship between LVDD and hypertension, and their relationship with LV systolic function. We also want to investigate if there is a relation between the arterial stiffness and LV structure and function in hypertensive patients [22, 23].

## The Assessment of LV Diastolic Function

LVDD is an important predictor of symptoms and clinical outcomes in patients with left ventricular systolic dysfunction (LVSD) [1, 2, 24]. Abnormalities of diastolic function in this population include an increase in LV filling pressures and LV volumes and impaired LV relaxation [25]. In a recent paper, “pre-clinical diastolic dysfunction” (PDD) has been defined as LVDD without congestive heart failure diagnosis and with normal systolic function [3]. Although invasive measures of LV relaxation and LV filling pressures (i.e., LV end-diastolic pressure) are considered to be the “gold standards” for the assessment of diastolic function, they are not performed in stable outpatients with LVSD. Pulsed-wave Doppler (PWD)-derived transmitral inflow patterns are commonly used for assessment of LVDD [26-28].

However, patients with LVSD often have variability in PWD-derived indices of LVDD due to increases in LA pressure, so they are preload-dependent [29, 30]. Tissue Doppler imaging (TDI)-derived early diastolic mitral annular velocity (E’) and color M-mode (CMM) imaging flow propagation velocity (Vp) have been reported to be less load-dependent methods to assess LV relaxation [31-33].

In addition, the diastolic intraventricular pressure gradient (IVPG) can be derived from CMM imaging by mathematical calculations of the spatio-temporal distribution of the early diastolic blood flow velocities into the LV cavity [34, 35]. IVPG is reported to be a relatively preload-independent measurement of LV relaxation [36, 37]. The evaluation of diastolic function by TDI and CMM-derived measurements to assess diastolic function in patients with LVSD has not been well evaluated. The measurement of these myocardial parameters could be of great importance in patients with HFpEF because these echocardiographic indices well describe the multidirectional function of whole LV myocardium, thereby allowing a detection of LV global function affections that is associated with a worse in symptomatic status of these patients.

## LVDD and Prognosis

**Figure 3:**
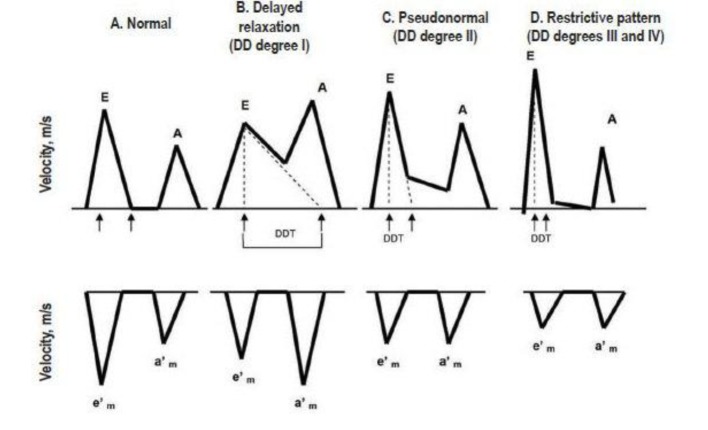
Different patterns of LVDD by transmitral flow pattern (upper) and tissue Doppler at mitral annulus level (lower). DDT: diastolic deceleration time, E and e’_m_: early ventricular filling; A and a’_m_: atrial contraction.

LVDD develops early in most cardiac diseases and leads to the elevation of LV filling pressures. Therefore, echocardiographic measurements of diastolic function provide important prognostic information. E/A ratios (early phase of atrial filling phases) reflect the compensatory increase in the late (atrial) filling phase when hypertrophic LV fails to relax normally during diastole. The result is that the E/A ratio on the mitral Doppler pattern decreases or reverses. However, when LV hypertrophy increases and wall fibrosis develops, LV chamber compliance decreases and the E wave again rises. Thus, it becomes difficult to separate E/A ratios that are truly normal from pseudonormal patterns of mitral inflow. Doppler tissue can measure the actual velocity of tissue relaxation of the mitral valve annulus or the posterior wall. Clinical studies have shown the association of short mitral diastolic deceleration time (DDT) with heart failure, death and hospitalizations in patients with acute myocardial infarction [38-54] (Fig. 3). Diastolic measurements provide incremental information to wall motion score index, as assessed by a recent meta-analysis of 12 post-acute myocardial infarction studies involving 1,286 patients [55]. Similar findings were reported in patients with ischemic or dilated cardiomyopathy, including atrial fibrillation. Pulmonary venous velocities [42, 56-58] and Vp [59] were less frequently performed but were still predictive of clinical events [60, 61]. Given the variability in measuring DT, Vp, and pulmonary venous flow velocity duration, recent studies have examined the prognostic value of E/E^1^. Several studies [62-74] have shown that E/E^1^ is highly predictive of adverse events after acute myocardial infarction and in hypertensive cardiomyopathy, severe secondary mitral regurgitation (MR), end-stage renal disease, atrial fibrillation, and cardiomyopathic disorders. The E/E^1^ ratio is among the most reproducible echocardiographic parameter to estimate pulmonary capillary wedge pressure and is the preferred prognostic parameter in many cardiac conditions. While all Doppler parameters abovementioned estimate LVDD at moment of the performance, left atrial structural and functional remodeling parameters have been proposed as a barometer of diastolic burden over time and as predictor of common cardiovascular outcomes, such as atrial fibrillation, stroke, congestive heart failure, and cardiovascular death [75, 76].

### Aortic stiffness

Aortic stiffening is increasingly recognized as an early marker of future cardiovascular disease and mortality among general population and hypertensive patients [77-80].

Classic risk scores may underestimate the risk of cardiovascular events in specific groups suitable for early prevention, such as asymptomatic hypertensive patients, often being wrongly classified as at low or moderate risk. Arterial stiffness is the most important determinant of increasing systolic and pulse pressures in ageing societies, thus giving a relevant contribution to incidence of stroke and myocardial infarction. Arterial stiffness has a predictive value for cardiovascular events, beyond classical cardiovascular risk factors and gives direct evidence of target organ damage, being itself a “tissue biomarker” [81]. Increased aortic stiffness is considered to be involved into artery’s ageing process [79], it determinates an increase in systolic blood pressure (SBP) and a decrease in diastolic blood pressure (DBP) resulting in an augmented LV workload and reduced perfusion of the coronary arteries during diastole [81]. Increased aortic stiffness, a major mechanical factor predicting CV risk, has been well identified as playing a role in metabolic syndrome. Its age progression seems to be proportional to the number of risk factors involved in metabolic syndrome and is responsible for increased SBP and decreased DBP with increasing age, the principal hallmarks of hypertension in the elderly [78]. Aortic stiffness is assessed by PWVg measured using two-dimensional (2D) echocardiography. The PWVg is calculated between the aortic valve and right common femoral artery by dividing the straight line distance between the two by the transit time. The distance is assessed using a tape measure located at the same place as the ultrasound probe. The transit time was defined as the difference between two intervals of time using the Doppler method [82]. PWVg is a simple, accurate, noninvasive means for the determination of large-artery stiffness, and it does not require dedicated equipment because it is performable by common echocardiographic machine, does not need training and is not time consuming [80]. Aortic PWV is considered an intrinsic measure of arterial stiffness according to the Moens-Korteweg equation where PWV is proportional to the square root of the incremental elastic modulus, of the vessel wall given constant ratio of wall thickness, to vessel radius and blood density, assuming that the artery wall is isotropic and experiences isovolumetric change with pulse pressure [80]. So PWV is related to the pulsatile component of LV afterload and is linked to prognostically adverse cardiac phenotype, including depressed LV systolic function [83, 84]. The strong relation between PWV and LV is also true because the pulse wave is generated by the contracting heart, and aortic PWV might be partially determined by enhanced myocardial performance, with a shortened LV ejection time, in young subjects [82]. If the initial speed of the pressure wave is mainly determined by the velocity of myocardial shortening [85] and LV ejection time is related with shortening velocity [86], we can assess that myocardial function and pulse pressure influence each other. The relation between aortic PWV and LV mass and function have been understudied until recent years. However, it may support a better comprehension of mechanism of HFpEF, a condition associated with high morbidity and mortality, whose prevalence is increasing, and is common among postmenopausal women. There are sex differences in aortic stiffness and its influence on left ventricle mass, geometry and function, postmenopausal women display increased arterial and LV stiffening, so LVDD [87, 88]. In addition, the association of increased arterial stiffness with mortality is almost two-fold higher in women than in men [89].

## Conclusions

Although LVDD remains poorly understood, it has an important clinical significance. LV diastolic function is influenced by arterial stiffness, changing the different components of its load, modulates LV structure and function. In patients affected by arterial hypertension, the pressure-volume loop shifts to the upper right side, therefore, pulmonary congestion is induced by a significant increase in LV end-diastolic pressure. To preserve LV ejection fraction, the pressure-volume loop shifts right due to a preload increase. Therefore, the LV pressure-volume loop operates on the ascending section of the end-diastolic pressure-volume curve, consequently causing end-diastolic pressure to arise. There is a strong relationship between arterial stiffness and the diastolic properties of the left ventricle. With increasing age, arterial stiffness becomes related to LV hypertrophy and to impaired LV diastolic function [77, 78, 90]. Aortic stiffness is related to electrocardiographically determined LVH in patients with hypertension [79]. Aortic PWVg is widely used to estimate arterial stiffness as elastic properties of the arterial tree and it is a strong predictor of cardiovascular outcomes in different clinical settings, including essential hypertension [91, 92]. Large artery stiffness is related to an array of functional and structural changes of the left ventricle [88, 93-95]. An elevated aortic impedance is a major stimulus for the development of LV unfavorable changes in diastolic function and a significant increase in LV mass. The more possible complete study of LV diastolic function and arterial stiffness leads to a complete detection of different mechanisms involved into complex functional and structural modifications of the hypertensive heart.
